# Protein kinase C in human renal cell carcinomas: role in invasion and differential isoenzyme expression

**DOI:** 10.1054/bjoc.1999.1043

**Published:** 2000-02-01

**Authors:** R Engers, S Mrzyk, E Springer, D Fabbro, G Weissgerber, C D Gerharz, H E Gabbert

**Affiliations:** Institute of Pathology, Heinrich-Heine-University, Moorenstr. 5, Duesseldorf, 40225, Germany; Oncology Research, Novartis Pharma 176, Basel, Switzerland

**Keywords:** protein kinase C, renal cell carcinoma, invasion

## Abstract

The role of protein kinase C (PKC) in in vitro invasiveness of four different human renal cell carcinoma (RCC) cell lines of the clear cell type was investigated. Different PKC-inhibitors markedly inhibited invasiveness of the highly invasive cell lines, suggesting an invasion-promoting role of PKC in human RCC. Analysis of PKC-isoenzyme expression by protein fractionation and immunoblotting revealed that all cell lines expressed PKC-α, -ɛ, -ζ, -μ and -ι as known from normal kidney tissue. Interestingly, PKC-δ, known to be expressed by normal kidney epithelial cells of the rat, was absent on protein and RNA levels in all RCC cell lines investigated and in normal human kidney epithelial cells. PKC-ɛ expression levels correlated positively with a high proliferation activity, but no obvious correlation between expression levels of distinct PKC-isoenzymes and in vitro invasiveness was observed. However, by immunofluorescence microscopy, membrane localisation of PKC-α and PKC-ɛ reflecting activation of the enzymes, was associated with a highly invasive potential. In conclusion, our results suggest a role for PKC in invasion of human RCCs and might argue in favour of a particular role of PKC-α and PKC-ɛ. Our results further suggest that organ-specific expression patterns of PKC-isoenzymes are not necessarily conserved during evolution. © 2000 Cancer Research Campaign


British Journal of Cancer (2000) 82(5), 1063–1069
© 2000 Cancer Research Campaign
DOI: 10.1054/ bjoc.1999.1043, available online at http://www.idealibrary.com on IDEAL®

### Protein kinase C in human renal cell carcinomas: role in invasion and differential isoenzyme expression

**R Engers[1], S Mrzyk[1], E Springer[1], D Fabbro[2], G Weissgerber[2], CD Gerharz[1] and HE Gabbert[1]**

[1]Institute of Pathology, Heinrich-Heine-University, Moorenstr. 5, 40225 Duesseldorf, Germany; [2]Oncology Research, Novartis Pharma 176, Basel, Switzerland

**Summary** The role of protein kinase C (PKC) in in vitro invasiveness of four different human renal cell carcinoma (RCC) cell lines of the clear cell type was investigated. Different PKC-inhibitors markedly inhibited invasiveness of the highly invasive cell lines, suggesting an invasion-promoting role of PKC in human RCC. Analysis of PKC-isoenzyme expression by protein fractionation and immunoblotting revealed that all cell lines expressed PKC-α, -ε, -ζ, -μ and -ι as known from normal kidney tissue. Interestingly, PKC-δ, known to be expressed by normal kidney epithelial cells of the rat, was absent on protein and RNA levels in all RCC cell lines investigated and in normal human kidney epithelial cells. PKC-ε expression levels correlated positively with a high proliferation activity, but no obvious correlation between expression levels of distinct PKC-isoenzymes and in vitro invasiveness was observed. However, by immunofluorescence microscopy, membrane localisation of PKC-α and PKC-ε reflecting activation of the enzymes, was associated with a highly invasive potential. In conclusion, our results suggest a role for PKC in invasion of human RCCs and might argue in favour of a particular role of PKC-α and PKC-ε. Our results further suggest that organ-specific expression patterns of PKC-isoenzymes are not necessarily conserved during evolution. © 2000 Cancer Research Campaign

**Keywords:** protein kinase C; renal cell carcinoma; invasion

Renal cell carcinoma (RCC) of the clear cell type is the most common malignant tumour of the kidney and has a very poor prognosis. Approximately one-third of the patients with RCC develop metastasis and systemic treatment using chemotherapy and/or cytokines proved to be rather ineffective (Mulders et al, 1997). Cytogenetic and molecular investigations of sporadic RCC revealed frequent chromosome 3p deletions and additional loss of heterozygosity on chromosomes 5, 11 and 17 (Kovacs et al, 1988; Anglard et al, 1991; Morita et al, 1991; Druck et al, 1995). Although these studies led to the identification of different genes, which are thought to be involved in carcinogenesis of RCC, the molecular mechanisms regulating aggressive properties of RCCs, such as invasion and metastasis, are still poorly understood.

Protein kinase C (PKC) reconstitutes a family of at least 11 related isoenzymes, which plays a major role in intracellular signal transduction and is thought be involved in cancer biology (Blobe et al, 1994; Stabel et al, 1994; Liu et al, 1996). According to their structural and biochemical characteristics, PKC-isoenzymes can be subdivided into three major groups: the conventional or classical PKC-isoenzymes (α, $\beta_I$, $\beta_{II}$, γ), the novel PKC-isoenzymes (δ, ε, η(L), θ, μ), and the atypical PKC-isoenzymes (ζ, ι(λ)). In general the biological effects of PKC seem to be tumour- or even cell line-specific rather than unique. Thus, PKC is thought to act as an oncogene in breast cancer, whereas in colon cancer it was suggested to act as an anti-oncogene (Blobe et al, 1994). These differences may at least in part be explained by the fact that different tissues exhibit different expression patterns of PKC-isoenzymes, and that the subcellular distribution of a particular PKC-isoenzyme varies depending on the cell type and conditions analysed (Blobe et al, 1994; Jaken, 1996). Moreover, it was shown by transfection experiments that overexpression of the same PKC-isoenzyme may result in opposite cell biological effects, depending on the cell line used (Borner et al, 1995; Goldstein et al, 1995). Although the expression of PKC-isoenzymes has been investigated in different rodent and murine tissue types and some cell lines of diverse species (Wetsel et al, 1992; Bareggi et al, 1995), little is known about the differential expression in human tumours and specific functional implications of each isoenzyme in tumour cell behaviour. The fact that PKC-isoenzymes differ, e.g. in organ-specific expression patterns, subcellular distribution upon overexpression in fibroblasts, substrate specificity and oncogenic potential, suggests specific biological roles for each PKC-isoenzyme. However, these roles appear to be at least partly cell type-specific (Jaken, 1996).

Studies on primary renal proximal tubule epithelial cells (RPTE), from which RCCs of the clear cell type are histogenetically derived, suggest that PKC might play a regulatory role for the biological properties of RCCs. In these studies primary RPTE as well as E1A-immortalized and SV40-transformed RPTE of the rat were compared for the expression and distribution of PKC-isoenzymes (Dong et al, 1994). Thereby, all cell lines were found to express PKC-isoenzymes α, δ, ε and ζ, and increased basal degradation rates of PKC-α were only observed in proliferating and oncogene-altered cells. Moreover, PKC-α was found to be partly associated with cell–cell junctions in primary RPTE, but this association diminished or disappeared in E1A-immortalized or SV40-transformed RPTE respectively (Dong et al, 1993).

In the present study, we analysed the effects of different PKC-inhibitors on invasion of human RCC in vitro, and in vitro invasiveness was compared to distinct expression levels and intracellular distribution patterns of PKC-isoenzymes.

*Received 7 July 1999*
*Revised 13 October 1999*
*Accepted 18 October 1999*

*Correspondence to:* R Engers

### MATERIALS AND METHODS

### Cell lines and cell culture conditions

The human RCC cell lines clearCa-5, clearCa-19, clearCa-28 and clearCa-39 were established from four different human RCCs of the clear cell type with histologically confirmed diagnosis as previously described (Gerharz et al, 1993). All cell lines were maintained in Dulbecco's modified Eagle medium (DMEM; Gibco, Karlsruhe, Germany) supplemented with 10% fetal calf serum (FCS) and antibiotics. Cell lines were incubated at 37°C in an atmosphere of 5% carbon dioxide.

### Growth characteristics

Since growth properties of the tumour cell lines might interfere with the chick heart invasion assay (CHIA) (see below), proliferation activities of all four cell lines were determined as previously described (Engers et al, 1994). Briefly, 15 replicate 25-$cm^2$ flasks were inoculated with $3 \times 10^5$ cells each. Cells from three culture flasks were harvested separately each day for a period of 120 h and counted by means of a Neubauer chamber. Doubling times were determined during the exponential growth phase.

### PKC-inhibitors

In order to investigate the role of PKC in human RCC invasion, different PKC inhibitors were used. H7 and staurosporine were purchased from Sigma (Deisenhofen, Germany). Although not absolutely PKC-specific, these compounds have been used in the past in a lot of studies on PKC, but are known also to inhibit some other protein kinases with a similar potency. CGP 41251 and GF 109203X were kindly provided by Dr T Meyer, Ciba-Geigy (Basel, Switzerland) and Dr J Kirilowsky, Glaxo (Les Ulies, France), respectively. Although less potent than their progenitor staurosporine, both compounds exhibit a much higher selectivity for PKC (Meyer et al, 1989; Toullec et al, 1991). However, not all PKC-isoenzymes are affected by these inhibitors. Stock solutions of each PKC-inhibitor were prepared with dimethylsulphoxide (DMSO) and diluted to final concentrations with standard growth medium.

In order to exclude that possible effects of these inhibitors on invasion and migration were mimicked by effects on cell proliferation or toxicity, in initial studies different concentrations of these compounds were investigated for their effect on cell growth and cell viability (data not shown). For each compound the respective maximal non-toxic concentration which did not interfere with cell proliferation was determined and chosen for subsequent experiments. These concentrations were: 5 nM (CGP 41251), 10 nM (staurosporine), 500 nM (GF 109203X) and 100 μM (H7).

### Chick heart invasion assay

In vitro invasiveness was determined by means of the CHIA as described (Engers et al, 1999). This assay was chosen because it closely resembles the complex in vivo mechanism of tumour invasion into a three-dimensional host tissue (Mareel, 1980; Engers et al, 1999). Heart fragments, dissected from a 9-day-old embryonic chick, were precultured on a Gyratory shaker for 4 days in order to round up and form spheroids. Spheroids with a diameter of 0.4 mm were selected and brought in contact with tumour cell aggregates (0.2 mm diameter) on top of a semisolid agar. After attachment the confronting pairs were individually transferred into fluid culture medium (either standard growth medium or medium supplemented with PKC-inhibitors) for further incubation. After incubation for the respective indicated periods of time, five confronting pairs each were fixed, followed by embedding in paraffin and complete serial sectioning. The three-dimensional extent of invasion was quantified by estimating the volume of host tissue that had been replaced by the tumour cells according to the following scale: 0–5%, 6–25%, 26–50%, 51–75% and 76–100%.

PKC-inhibitors were used at final concentrations of 100 μM (H7), 10 nM (staurosporine), 5 nM (CGP 41251) and 0.5 μM (GF 109203X). DMSO did not affect invasion at the concentrations used in this study (data not shown).

### Protein fractionation and immunoblotting

Subconfluent monolayers were lysed using an ice-cold buffer that was 20 mM Tris–HCl (pH 7.4), 2 mM EGTA, 2 mM disodium–calcium–EDTA, 6 mM mercaptoethanol, 2 μg $\mu l^{-1}$ aprotinin and 2 μg $ml^{-1}$ leupeptin. Lysates were sonicated on ice for ten cycles, centrifuged (13 000 rpm, 4°C) and supernatants were collected as soluble fraction. The remaining pellets were resuspended in lysis buffer, supplemented with 1% NP-40 and sonicated for another ten cycles. After centrifugation (13 000 rpm, 4°C) supernatants were collected as particulate fractions. Equal amounts of protein were separated by sodium dodecyl sulphate polyacrylamide gel electrophoresis (SDS-PAGE) and transferred to nitrocellulose membrane. Detection of PKC-α, -$\beta_I$, -$\beta_{II}$, -γ, -δ, -ε, η(L), -θ, -μ and -ζ was performed by means of polyclonal antibodies, generated against isoenzyme-specific peptide sequences of the C-terminus as described (Marte et al, 1994; Johannes et al, 1995). Two different polyclonal antibodies against PKC-ι were purchased from Santa Cruz Biotechnology (Heidelberg, Germany) and Transduction Laboratories (Lexington, KY, USA). For detection a commercially available ECL-detection kit (Boehringer Mannheim, Mannheim, Germany) was used according to the manufacturer's instructions.

In case of PKC-δ additional total cell lysates were prepared from all cell lines and from normal human kidney epithelial cells using the same buffer, containing 1% NP-40, as mentioned above. As a positive control the rat rhabdomyosarcoma cell line BA-HAN-1C (Gabbert et al, 1988) was used.

### Immunocytochemistry

Intracellular distribution of PKC-isoenzymes was determined by means of immunofluorescence microscopy which was essentially performed as previously described (Engers et al, 1994). Primary antibodies used were polyclonal isoenzyme-specific antibodies against PKC-α, -δ, -ε, or -ζ from Santa Cruz (Heidelberg, Germany). Two different polyclonal antibodies against PKC-ι were purchased from Santa Cruz Biotechnology (Heidelberg, Germany) and Transduction Laboratories (Lexington, KY, USA). A monoclonal antibody against PKC-μ was kindly provided by Dr FJ Johannes (Stuttgart, Germany) (Johannes et al, 1994). Secondary antibodies were fluorescein isothiocyanate (FITC)-labelled anti-rabbit or anti-mouse antibodies, purchased from Dako (Hamburg, Germany).

 *© 2000 Cancer Research Campaign*

### Southern blot analysis

Chromosome 3p deletions are a common characteristic of clear cell RCCs (Kovacs et al, 1988). In order to determine whether the observed absence of PKC-δ expression on protein and RNA levels in our RCC cell lines (see below) was caused by a deletion of the PKC-δ gene, which is located on the short arm of chromosome 3, Southern blot analysis was performed. Total genomic DNA from tumour cell lines and from normal kidney tissue, which served as control, was prepared according to standard protocols. Four micrograms of either *Bam*HI, *Eco*RI, *Xho*I, *Pst*I, or *Hin*dIII-digested DNA were run on a 1% agarose gel and were alkaline blotted overnight onto a Gene Screen Plus membrane according to the manufacturer's instructions (NEN Lifescience, Boston, MA, USA). Blots were probed with a cloned, full-length 2 kb rat PKC-δ cDNA fragment, kindly provided by Dr FJ Mushinski (Bethesda, MD, USA), using the Rediprime Kit (Amersham Life Science, Little Chalfort, UK). Hybridisation was performed overnight in sodium phosphate-buffered formamide solution (50% formamide, 5 × SSC (saline–sodium citrate), 1% SDS, 2 × Denhardt's, 5% dextran sulphate, 0.2 mg $ml^{-1}$ salmon sperm DNA) and after several washing steps exposed to KODAK XAR films overnight.

### RESULTS

### In vitro invasiveness and effects of different PKC-inhibitors

In the CHIA, imitating the complex mechanism of tumour cell invasion into a three-dimensional host tissue, major differences in in vitro invasiveness became evident among the four human RCC cell lines (Figure 1A). ClearCa-5 and clearCa-19 were highly invasive, invading the host tissue by 76–100% after 10 days of incubation. In contrast, clearCa-28 and clearCa-39 exhibited only low invasive capacities, the maximal extent of invasiveness not exceeding 5% of the host tissue after 10 days of incubation.

Since differences in cell proliferation might contribute to differences in in vitro invasiveness after long time of incubation, growth properties of all four cell lines were determined. Although marked differences in cell growth were seen among the four cell lines, no correlation became evident between growth activity and in vitro invasiveness. Thus, the highly invasive cell line clearCa-19 exhibited a markedly lower proliferation activity (mean doubling time 74.6 h) than the low invasive cell line clearCa-28 (mean doubling time 34.7 h). These results exclude that the observed differences in invasion among the different cell lines resulted simply from differences in proliferation. The mean doubling times for clearCa-5 and clearCa-39 were 27.5 h and 121.4 h respectively.

In order to investigate the functional role of PKC in human RCC invasion, the effects of different PKC-inhibitors were investigated. Whereas in vitro invasiveness of the weakly invasive cell lines clearCa-28 and clearCa-39 was not affected by either compound (data not shown), marked anti-invasive effects were observed in the highly invasive cell lines clearCa-5 and clearCa-19 (Figure 1B). Over a time course of 7 days, H7 exhibited the most pronounced effects, inhibiting invasion of both clearCa-5 and clearCa-19 by approximately 70% and 90%, respectively, compared to non-treated control cells. Similar to H7, both staurosporine and the specific PKC-inhibitor CGP 41251 markedly inhibited invasion of clearCa-5 and clearCa-19, but the effects were less pronounced at the concentrations used. In contrast to CGP 41251, another staurosporine-derived specific PKC-inhibitor, GF 109203X, inhibited invasion exclusively in clearCa-5 and only during the first 4 days.

### Immunoblotting

To investigate, whether differences in invasion between the four human RCC cell lines were correlated with differences in PKC-isoenzyme expression, immunoblot analyses were performed. Since activation of PKC-isoenzymes results in a redistribution of the proteins and association with intracellular membranes, proteins were extracted from both soluble (cytosolic) and particulate (membrane) fractions. All cell lines expressed PKC-α, -ε, -μ, -ζ and -ι (Figure 2A), whereas PKC-$\beta_I$, -$\beta_{II}$ -δ, -γ, -η(L) and -θ proved to be absent as confirmed by RT-PCR and subsequent isoenzyme-specific oligonucleotide hybridisation (data not shown). PKC-α was either predominantly or exclusively expressed in the soluble, PKC-ε predominantly in the particulate fractions of all four RCC cell lines. Except for PKC-μ in clearCa-19 and PKC-ζ in clearCa-39, PKC-μ, -ζ and -ι were not preferentially expressed in any of these fractions. Moreover, no obvious association was found between in vitro invasiveness and the expression levels of any PKC-isoenzyme. However, the strongest expression levels of PKC-ε were found in clearCa-5 and clearCa-28, and thus correlated with a high proliferation activity.

Interestingly, PKC-δ, known to be expressed by RPTE cells of the rat, was absent in all of our RCC cell lines as well as in normal human kidney epithelial cells, while the rat rhabdomyosarcoma cell line BA-HAN-1C (Gabbert et al, 1988), used as a positive control, was positive (Figure 2B). This result cannot be attributed to species specificity of the antibody, because a polyclonal antibody, recognising both human and rat PKC-δ, was used.

### Intracellular distribution of PKC-isoenzymes

Since isoenzyme-specific subcellular distribution patterns of PKC are indicative for isoenzyme-specific functions (Goodnight et al, 1995), we investigated by immunofluorescence microscopy whether differences in invasion between our human RCC cell lines were associated with distinct intracellular distribution patterns of individual PKC-isoenzymes (Figure 3).

PKC-α was diffusely expressed in the cytoplasm of all cell lines and in association with cell membranes, when cell–cell contacts were established. Interestingly, the strongest association of PKC-α with cell–cell contacts, indicative for activation of PKC-α (Goodnight et al, 1995), was observed in cell lines with a highly invasive potential (clearCa-5 and clearCa-19), while in the low invasive cell lines membrane-association of PKC-α was either found to a markedly lower extent (clearCa-28) or was entirely absent (clearCa-39). Additional expression patterns of PKC-α such as a dotted staining throughout the cytoplasm and a focal enhancement in the perinuclear zone, indicating an association with the endoplasmic reticulum and the Golgi apparatus, respectively (Goodnight et al, 1995), did not correlate with invasive properties.

Similar to PKC-α the expression pattern of PKC-ε correlated with in vitro invasiveness, as localisation at cell membranes, reflecting activation of the enzyme (Goodnight et al, 1995), was only detected in the highly invasive cell lines clearCa-5 and clearCa-19. PKC-ε was also expressed in a punctated pattern throughout the cytoplasm

© 2000 Cancer Research Campaign

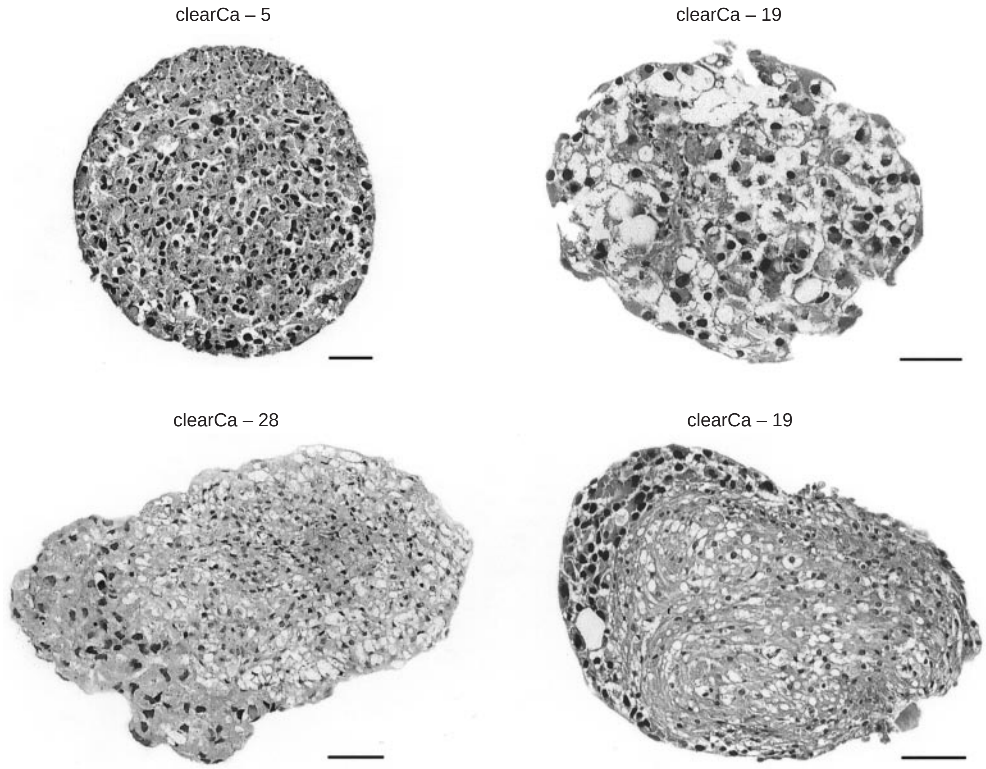

**A**

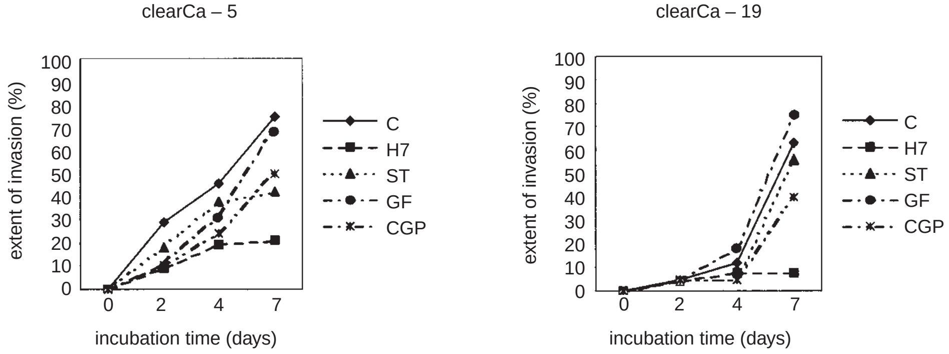

**B**

**Figure 1** In vitro invasiveness of human RCC cell lines as determined by the CHIA and effects of different PKC-inhibitors. (**A**) In standard growth medium clearCa-5 and clearCa-19 almost completely replaced the host tissue by invasion after 10 days of incubation, while clearCa-28 and clearCa-39 exhibited very low invasive capacities, the maximal extent of invasion not exceeding 5% of the host tissue after the same time of incubation. Results are representative for two independent experiments, each of which with 5 different spheroids per cell line. Scale bars, 50 μm. (**B**) Effects of different PKC-inhibitors on invasion of human RCC cell lines in the CHIA during a time course of 7 days. The extent of invasion is indicated as % of the host tissue which was replaced by tumour cell invasion. C: control, H7: H7, ST: staurosporine, GF: GF 109203X, CGP: CGP 41251. Results are representative for two independent experiments, each of which with five different spheroids per cell line, compound and day

(all cell lines), diffusely in the cytoplasm (clearCa-5, clearCa-19 and clearCa-28), and in the nucleus (clearCa-19). For these localisations, however, no correlation with invasion was found.

Expression patterns of PKC-ζ and PKC-μ were partly heterogeneous, cell line- and isoenzyme-specific. An association with in vitro invasiveness was not observed. In contrast to immunoblot analysis, PKC-ι was not detectable by immunocytochemistry in any of the cell lines, although two different antibodies and different concentrations of the antibodies were used.

In some cases cell membrane localisation of distinct PKC isoenzymes was not observed by immunofluorescence microscopy, although clear signals were seen in the respective particulate fractions by immunoblotting. These results, however, are not necessarily contradictory, because particulate fractions not only harbour proteins associated with the cell membrane, but also proteins associated with intracellular membranes such as from the Golgi apparatus or the endoplasmic reticulum.

 *© 2000 Cancer Research Campaign*

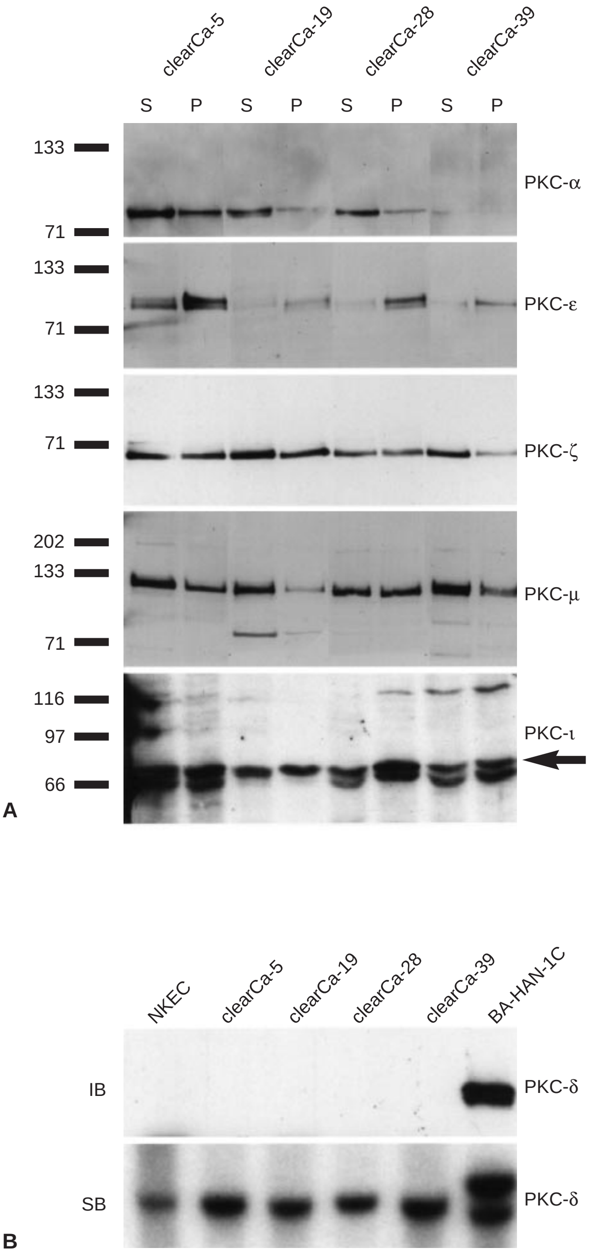

**Figure 2** PKC-isoenzymes in clearCa-5, clearCa-19, clearCa-28 and clearCa-39. (**A**) Expression levels of PKC-isoenzymes separated for both soluble (s) and particulate (p) fractions as determined by immunoblotting. PKC-$\beta_I$, -$\beta_{II}$, -γ, -δ, -η(L) and -θ were not expressed. (**B**) Immunoblot (IB) and Southern blot analysis (SB) for PKC-δ in the same human RCC cell lines, normal human kidney epithelial cells (NKEC) and the rat rhabdomyosarcoma cell line BA-HAN-1C which served as a positive control. Immunoblots were performed from total cell lysates. The southern blot shown was performed using *Bam*HI-digested DNA. Double bands in BA-HAN-1C result from an additional *Bam*HI restriction site in rat PKC-δ when compared to human PKC-δ. Results are representative for three independent experiments

### Expression analysis for PKC-δ

Although in rat kidneys PKC-δ is known to be expressed by RPTE cells, from which RCCs of the clear cell type are histogenetically derived (Dong et al, 1994), PKC-δ was absent in all human RCC cell lines investigated, as shown by immunoblotting and immunofluorescence microscopy. This lack of PKC-δ expression was confirmed by immunoprecipitation/immunoblotting and by RT-PCR and PKC-δ-specific oligonucleotide hybridisation (data not shown). The PKC-δ gene is located on chromosome 3p, but its exact position is still unknown (Huppi et al, 1994). Since deletions on chromosome 3p are observed in more than 90% of RCCs of the clear cell type (Kovacs et al, 1988), we investigated whether the observed lack of PKC-δ expression in our RCC cell lines was due to a deletion of the PKC-δ gene. Southern blot analysis revealed, however, that all cell lines as well as normal human kidney epithelial cells harboured the PKC-δ gene (Figure 2B). These results indicate that (1) the PKC-δ gene is not commonly deleted in human RCCs of the clear cell type and (2) that in contrast to rat kidney epithelial cells, human kidney epithelial cells lack PKC-δ expression on the protein and mRNA level.

### DISCUSSION

In the present study we characterised four different human RCC cell lines of the clear cell type for their invasive potential in vitro and investigated the effects of different PKC-inhibitors on human RCC invasiveness. At concentrations which did not affect cell growth, both non-selective and selective PKC-inhibitors exhibited marked anti-invasive effects in the highly invasive cell lines, clearCa-5 and clearCa-19, whereas invasion of the weakly invasive cell lines clearCa-28 and clearCa-39 was not affected. These results suggest an invasion-promoting role of PKC in human RCC as was also suggested for other tumour types, in which PKC-inhibitors of other selectivity than GF 109203X and CGP 41251 were used (Schwartz et al, 1993; Mapelli et al, 1994; Hoelting et al, 1996). Differences in the extent of inhibition of invasion among the different PKC-inhibitors might be explained by known differences of the inhibitors in: (1) their specificities for the PKC family, (2) their specificities for distinct PKC-isoenzymes and (3) by differences in the potency to inhibit distinct PKC-isoenzymes at certain subcellular localisations (Meyer et al, 1989; Toullec et al, 1991; Budworth and Gescher, 1995). Thus, H7 is about four times more effective in inhibiting membrane-bound PKC than cytosolic PKC, whereas inhibitory effects of CGP 41251 on cytosolic PKC are about 14 times stronger than on membrane-bound PKC. Moreover, differences in metabolic degradation during the incubation period might account for the reduced anti-invasive potencies of GF 109203X and CGP 41251 after 7 days of incubation, compared to H7 and staurosporine. Finally, the different PKC-inhibitors were used in our study at concentrations which did not affect cell proliferation. Therefore, in case distinct compounds inhibit proliferation at lower concentrations than invasion, their anti-invasive effects would have been underestimated.

We next investigated whether differences in invasion between the four different RCC cell lines were associated with differences in PKC-isoenzyme expression. By cell fractionation and immunoblotting all cell lines were shown to express PKC-isoenzymes α, ε, μ, ζ and ι. PKC-α was either predominantly or exclusively located in the soluble, PKC-ε in the particulate fraction, as was similarly reported for a breast cancer cell line (Disatnik et al, 1994). However, no preferential localisation of any other PKC-isoenzyme with one of these fractions was seen. When compared to cell biological properties, high expression levels of PKC-ε were correlated with a high proliferation activity, which is consistent with studies on fibroblasts, in which overexpression of PKC-ε induces cell growth and oncogenicity (Cacace et al, 1993). Our results

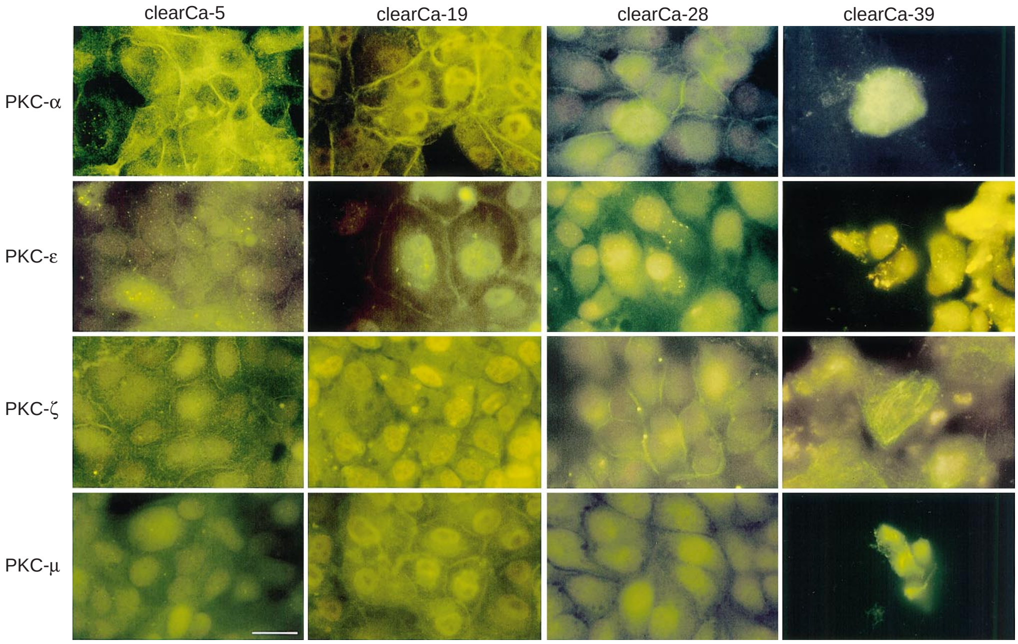

**Figure 3** Differential subcellular expression patterns of PKC-isoenzymes in human RCC cell lines as determined by immunofluorescence microscopy. Using a FITC-labelled secondary antibody, isoenzyme-specific localisations are indicated by a green fluorescence signal. Results are representative for three independent experiments. All pictures were taken by the same magnification. Scale bar, 25 μm therefore suggest that PKC-ε might also be involved in the regulation of proliferation in human RCCs.

In contrast to proliferation, no strict correlation was observed between the expression levels of distinct PKC-isoenzymes, as determined by immunoblotting, and in vitro invasiveness. This lack of correlation, however, does not necessarily rule out a role of PKC in RCC invasion, since not merely the expression levels, but also the subcellular distribution of PKC-isoenzymes is thought to be of major importance for their specific functional activities (Buchner et al, 1995; Goodnight et al, 1995; Kiley and Parker, 1995; Jaken et al, 1997). In our RCC cell lines the subcellular expression patterns of PKC-α, -ε, -μ and -ζ, as determined by immunofluorescence, proved to be partly isoenzyme- and cell line-specific. Nevertheless, a strong correlation between the subcellular expression patterns of both PKC-α and PKC-ε and in vitro invasiveness of human RCCs became evident. Thus, association of PKC-ε with the cell membrane and localisation with cell–cell contacts was exclusively restricted to the highly invasive cell lines and was completely absent in the low invasive cell lines. Similarly, the strongest association of PKC-α with cell–cell contacts was observed in cell lines with a high invasive potential, while in the low invasive cell lines membrane-association of PKC-α was either found to a markedly lower extent or was not detectable at all. Translocation of PKC-α and PKC-ε to the cell membrane has been shown to occur upon activation of the enzymes (Goodnight et al, 1995). Therefore, our results suggest a particular activation of PKC-α and PKC-ε in highly invasive RCC cell lines and hence a possible role of these PKC-isoenzymes in RCC invasiveness. These results are in line with the observation, that overexpression of PKC-α or PKC-ε induces a more aggressive or an oncogenic phenotype respectively (Cacace et al, 1993; Ways et al, 1995).

The observed expression pattern of PKC-isoenzymes (PKC-α, -ε, -μ, -ζ and -ι) in human RCC cell lines differs from that of other tumour types (Selbie et al, 1993; Disatnik et al, 1994; Mapelli et al, 1994) and therefore suggests tumour-specific rather than unique functions of PKC and PKC-isoenzymes. However, the presence of PKC-α, -ε and -ζ in RCCs is in line with studies on renal proximal tubule epithelial cells (RPTE) (Dong et al, 1994), from which RCCs of the clear cell type are histogenetically derived. PKC-μ and -ι are also known to be expressed by normal kidney tissue, though their expression has not yet been attributed to distinct cellular compartments of the kidney (Selbie et al, 1993; Johannes et al, 1994). Interestingly, PKC-δ, known to be expressed by both normal and transformed RPTE of the rat, was absent in all four RCC cell lines as well as in normal human kidney epithelial cells on protein and RNA levels. The PKC-δ gene is located on chromosome 3p, but its exact localisation is still unknown (Huppi et al, 1994). Since chromosome 3p deletions are found in almost all human RCCs of the clear cell type (Kovacs et al, 1988), it was tempting to speculate, whether the observed lack of PKC-δ expression in our tumour cell lines was due to a common deletion of the PKC-δ gene. However, by Southern blot analysis all cell lines as well as normal human kidney epithelial cells were shown to harbour the PKC-δ gene. These results indicate that: (1) the PKC-δ gene is not commonly deleted in human RCCs of the clear cell type, (2) that in contrast to rat kidney epithelial cells, human kidney epithelial cells lack PKC-δ expression on the protein and mRNA level, and hence that organ-specific expression patterns of PKC-isoenzymes are not necessarily conserved during evolution.

In conclusion, our results suggest a role for PKC in the regulation of human RCC invasiveness. The fact that distinct expression patterns of PKC-α and/or -ε were correlated with invasion and/or

*© 2000 Cancer Research Campaign*

proliferation might argue for a particular role of both PKC-isoenzymes in these biological properties. The elucidation of these roles will be subject of further studies.

### ACKNOWLEDGEMENTS

We thank Dr FJ Johannes for providing us with the PKC-μ antibody; Mrs A Florange-Heinrichs, Mrs S Sliwka, Mrs R Solf and Mr M Ringler for their excellent technical assistance. Some of the results are part of the medical thesis of S Mrzyk. The authors thank the grant sponsor: 'Dr Mildred Scheel Stiftung für Krebsforschung', grant number W 64/94/Ga.

### REFERENCES

Anglard P, Tory K, Brauch H, Weiss GH, Latif F, Merino MJ, Lerman MI, Zbar B and Linehan WM (1991) Molecular analysis of genetic changes in the origin and development of renal cell carcinoma. *Cancer Res* **51**: 1071–1077

Bareggi R, Grill V, Zweyer M, Narducci P and Martelli AM (1995) Distribution of the extended family of protein kinase C isoenzymes in fetal organs of mice: an immunohistochemical study. *Cell Tissue Res* **280**: 617–625

Blobe GC, Obeid LM and Hannun YA (1994) Regulation of protein kinase C and role in cancer biology. *Cancer Metastasis Rev* **13**: 411–431

Borner C, Ueffing M, Jaken S, Parker PJ and Weinstein IB (1995) Two closely related isoforms of protein kinase C produce reciprocal effects on the growth of rat fibroblasts. Possible molecular mechanisms. *J Biol Chem* **270**: 78–86

Buchner K (1995) Protein kinase C in the transduction of signals toward and within the cell nucleus. *Eur J Biochem* **228**: 211–221

Budworth J and Gescher A (1995) Differential inhibition of cytosolic and membrane-derived protein kinase C activity by staurosporine and other kinase inhibitors. *FEBS Lett* **362**: 139–142

Cacace AM, Guadagno SN, Krauss RS, Fabbro D and Weinstein IB (1993) The epsilon isoform of protein kinase C is an oncogene when overexpressed in rat fibroblasts. *Oncogene* **8**: 2095–2104

Disatnik MH, Winnier AR, Mochly RD and Arteaga CL (1994) Distinct responses of protein kinase C isozymes to c-erbB-2 activation in SKBR-3 human breast carcinoma cells. *Cell Growth Differ* **5**: 873–880

Dong L, Stevens JL, Fabbro D and Jaken S (1994) Transformation-sensitive localization of alpha-protein kinase C at cell–cell contacts in rat renal proximal tubule epithelial cells. *Cell Growth Differ* **4**: 793–798

Dong L, Stevens JL, Fabbro D and Jaken S (1994) Protein kinase C isozyme expression and down-modulation in growing, quiescent, and transformed renal proximal tubule epithelial cells. *Cell Growth Differ* **5**: 881–890

Druck T, Kastury K, Hadaczek P, Podolski J, Toloczko A, Sikorski A, Ohta M, Laforgia S, Lasota J, McCue P and ET AL (1995) Loss of heterozygosity at the familial RCC t(3;8) locus in most clear cell renal carcinomas. *Cancer Res* **55**: 5348–5353

Engers R, Gerharz CD, Donner A, Mrzyk S, Krause PR, Petek O and Gabbert HE (1999) In vitro invasiveness of human epithelioid-sarcoma cell lines: association with cell motility and inverse correlation with the expression of tissue inhibitor of metalloproteinases. *Int J Cancer* **80**: 406–412

Engers R, Gerharz CD, Moll R, Pohl A, Sarbia M and Gabbert HE (1994) Interclonal heterogeneity in a human epithelioid-sarcoma cell line (GRU-1). *Int J Cancer* **59**: 548–553

Gabbert HE, Gerharz CD, Biesalski HK, Engers R and Luley C (1988) Terminal differentiation and growth inhibition of a rat rhabdomyosarcoma cell line (BA-HAN-1C) in vitro after exposure to retinoic acid. *Cancer Res* **48**: 5264–5269

Gerharz CD, Moll R, Storkel S, Ramp U, Thoenes W and Gabbert HE (1993) Ultrastructural appearance and cytoskeletal architecture of the clear, chromophilic, and chromophobe types of human renal cell carcinoma in vitro. *Am J Pathol* **142**: 851–859

Goldstein DR, Cacace AM and Weinstein IB (1995) Overexpression of protein kinase C beta 1 in the SW480 colon cancer cell line causes growth suppression. *Carcinogenesis* **16**: 1121–1126

Goodnight JA, Mischak H, Kolch W and Mushinski JF (1995) Immunocytochemical localization of eight protein kinase C isozymes overexpressed in NIH 3T3 fibroblasts. Isoform-specific association with microfilaments, Golgi, endoplasmic reticulum, and nuclear and cell membranes. *J Biol Chem* **270**: 9991–10001

Hoelting T, Duh QY, Clark OH and Herfarth C (1996) Tamoxifen antagonizes proliferation and invasion of estrogen receptor-negative metastatic follicular thyroid cancer cells via protein kinase C. *Cancer Lett* **100**: 89–93

Huppi K, Siwarski D, Goodnight J and Mischak H (1994) Assignment of the protein kinase C delta polypeptide gene (PRKCD) to human chromosome 3 and mouse chromosome 14. *Genomics* **19**: 161–162

Jaken S (1996) Protein kinase C isozymes and substrates. *Curr Opin Cell Biol* **8**: 168–173

Jaken S (1997) Protein kinase C intracellular binding proteins. In: *Protein Kinase C*, Parker PJ and Dekker LV (eds), pp. 179–188. Landes:

Johannes FJ, Prestle J, Eis S, Oberhagemann P and Pfizenmaier K (1994) PKCu is a novel, atypical member of the protein kinase C family. *J Biol Chem* **269**: 6140–6148

Johannes FJ, Prestle J, Dieterich S, Oberhagemann P, Link G and Pfizenmaier K (1995) Characterization of activators and inhibitors of protein kinase C mu. *Eur J Biochem* **227**: 303–307

Kiley SC and Parker PJ (1995) Differential localization of protein kinase C isozymes in U937 cells: evidence for distinct isozyme functions during monocyte differentiation. *J Cell Sci* **108**: 1003–1016

Kovacs G, Erlandsson R, Boldog F, Ingvarsson S, Muller BR, Klein G and Sumegi J (1988) Consistent chromosome 3p deletion and loss of heterozygosity in renal cell carcinoma. *Proc Natl Acad Sci USA* **85**: 1571–1575

Liu JP (1996) Protein kinase C and its substrates. *Mol Cell Endocrinol* **116**: 1–29

Mapelli E, Banfi P, Sala E, Sensi M, Supino R, Zunino F and Gambetta RA (1994) Effect of protein kinase C inhibitors on invasiveness of human melanoma clones expressing different levels of protein kinase C isoenzymes. *Int J Cancer* **57**: 281–286

Mareel MM (1980) Recent aspects of tumor invasiveness. *Int Rev Exp Pathol* **22**: 65–129

Marte BM, Meyer T, Stabel S, Standke GJ, Jaken S, Fabbro D and Hynes NE (1994) Protein kinase C and mammary cell differentiation: involvement of protein kinase C alpha in the induction of beta-casein expression. *Cell Growth Differ* **5**: 239–247

Meyer T, Regenass U, Fabbro D, Alteri E, Rosel J, Muller M, Caravatti G and Matter A (1989) A derivative of staurosporine (CGP 41 251) shows selectivity for protein kinase C inhibition and in vitro anti-proliferative as well as in vivo anti-tumor activity. *Int J Cancer* **43**: 851–856

Morita R, Ishikawa J, Tsutsumi M, Hikiji K, Tsukada Y, Kamidono S, Maeda S and Nakamura Y (1991) Allelotype of renal cell carcinoma. *Cancer Res* **51**: 820–823

Mulders P, Figlin R, Dekernion JB, Wiltrout R, Linehan M, Parkinson D, Dewolf W and Belldegrun A (1997) Renal cell carcinoma: recent progress and future directions. *Cancer Res* **57**: 5189–5195

Prestle J, Pfizenmaier K, Brenner J and Johannes FJ (1996) Protein kinase C mu is located at the Golgi compartment. *J Cell Biol* **134**: 1401–1410

Schwartz GK, Jiang J, Kelsen D and Albino AP (1993) Protein kinase C: a novel target for inhibiting gastric cancer cell invasion. *J Natl Cancer Inst* **85**: 402–407

Selbie LA, Schmitz PC, Sheng Y and Biden TJ (1993) Molecular cloning and characterization of PKC iota, an atypical isoform of protein kinase C derived from insulin-secreting cells. *J Biol Chem* **268**: 24296–24302

Stabel S (1994) Protein kinase C – an enzyme and its relatives. *Semin Cancer Biol* **5**: 227–284

Toullec D, Pianetti P, Coste H, Bellevergue P, Grand PT, Ajakane M, Baudet V, Boissin P, Boursier E, Loriolle F and ET A (1991) The bisindolylmaleimide GF 109203X is a potent and selective inhibitor of protein kinase C. *J Biol Chem* **266**: 15771–15781

Ways DK, Kukoly CA, Devente J, Hooker JL, Bryant WO, Posekany KJ, Fletcher DJ, Cook PP and Parker PJ (1995) MCF-7 breast cancer cells transfected with protein kinase C-alpha exhibit altered expression of other protein kinase C isoforms and display a more aggressive neoplastic phenotype. *J Clin Invest* **95**: 1906–1915

Wetsel WC, Khan WA, Merchenthaler I, Rivera H, Halpern AE, Phung HM, Negro VA and Hannun YA (1992) Tissue and cellular distribution of the extended family of protein kinase C isoenzymes. *J Cell Biol* **117**: 121–133

© 2000 Cancer Research Campaign 
